# Supplementary figures and images for: Antibody responses to gSG6-p1, AgSAP, and SAMSP1 following anopheline salivary exposure
Source: Parasit Vectors. 2025 Oct 27;18:429. doi: 10.1186/s13071-025-07072-8 (PMC12560312; doi:10.1186/s13071-025-07072-8)

# **SUPPLEMENTARY MATERIAL**


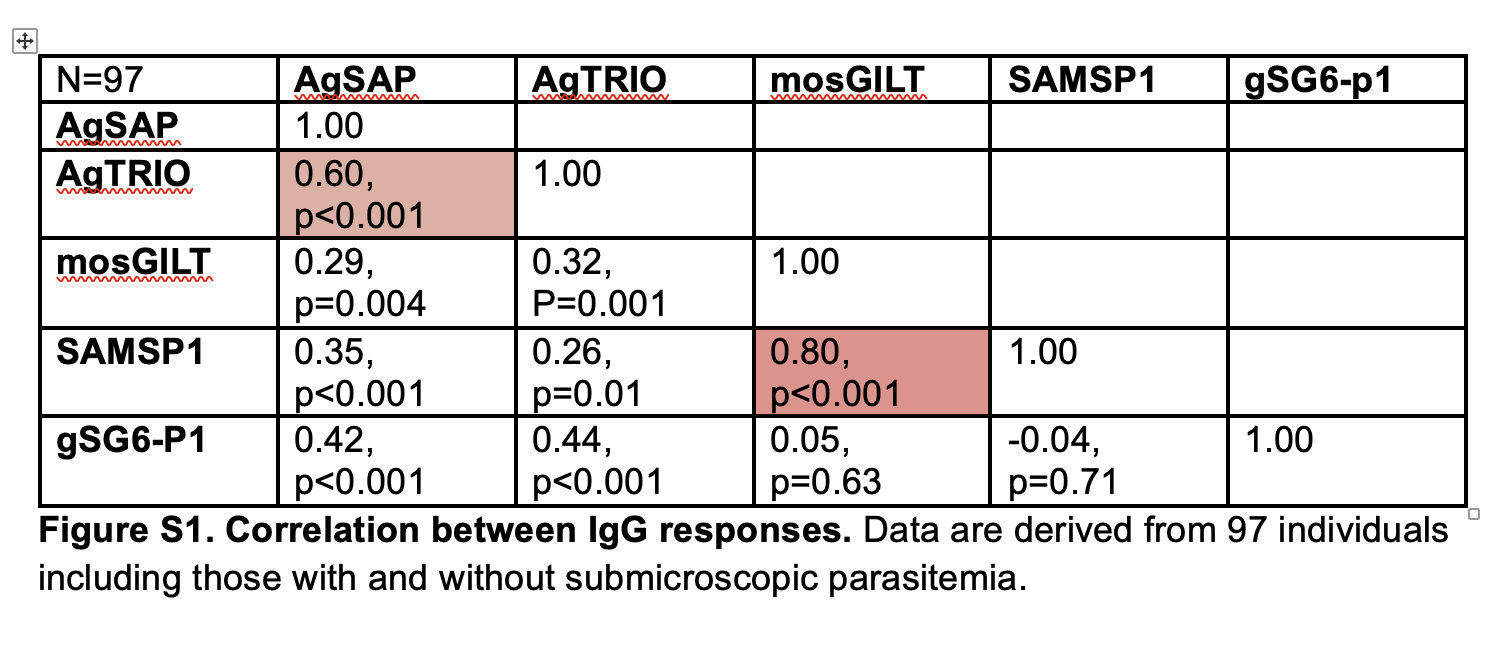


**
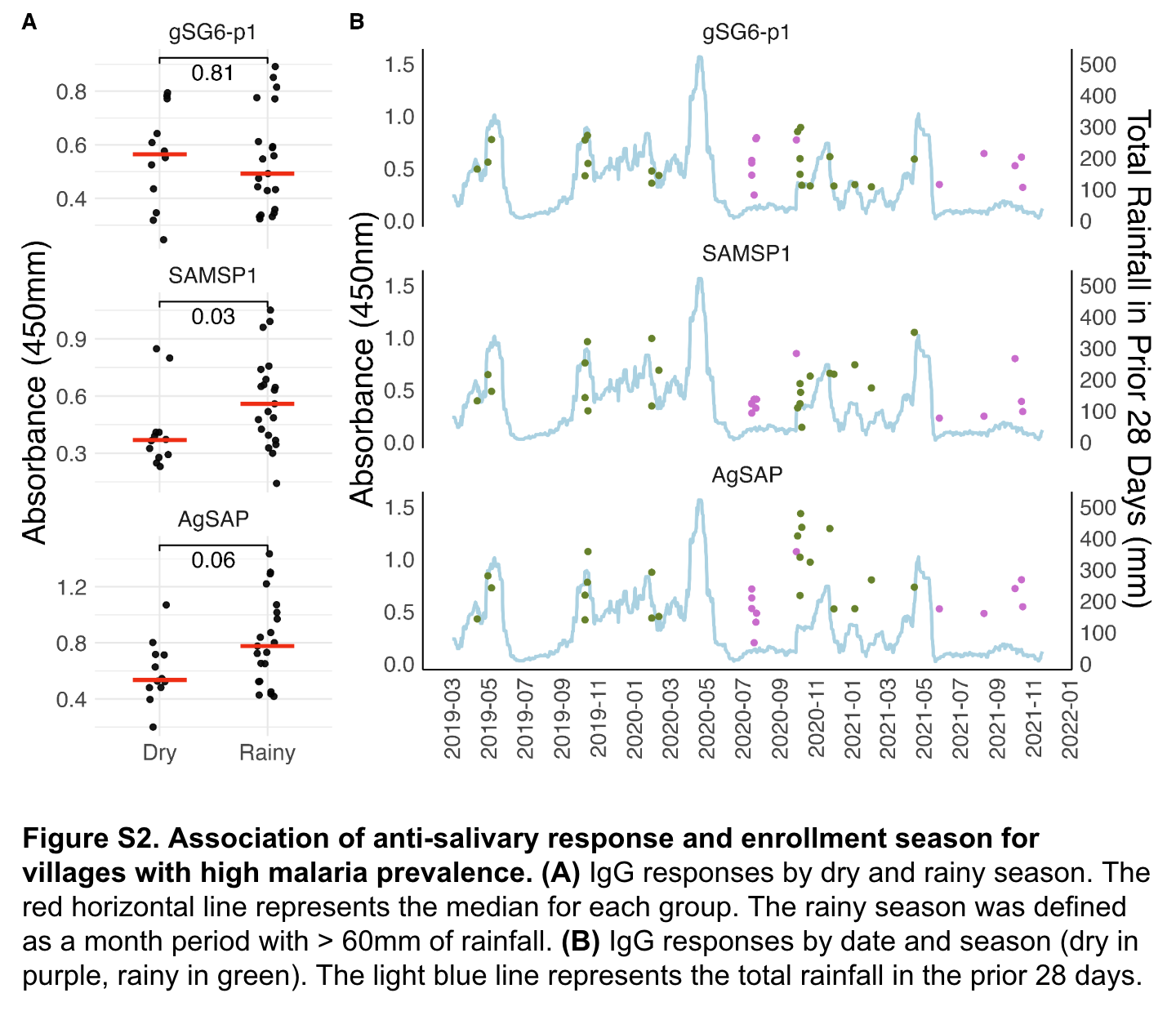
**

**
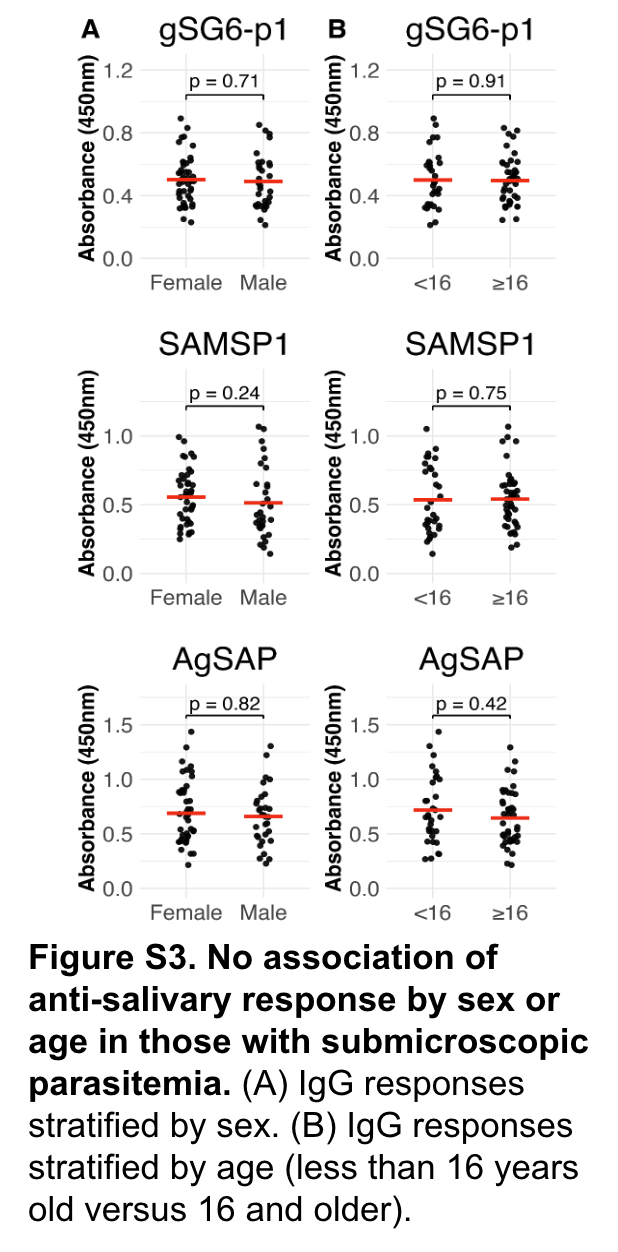
**

**
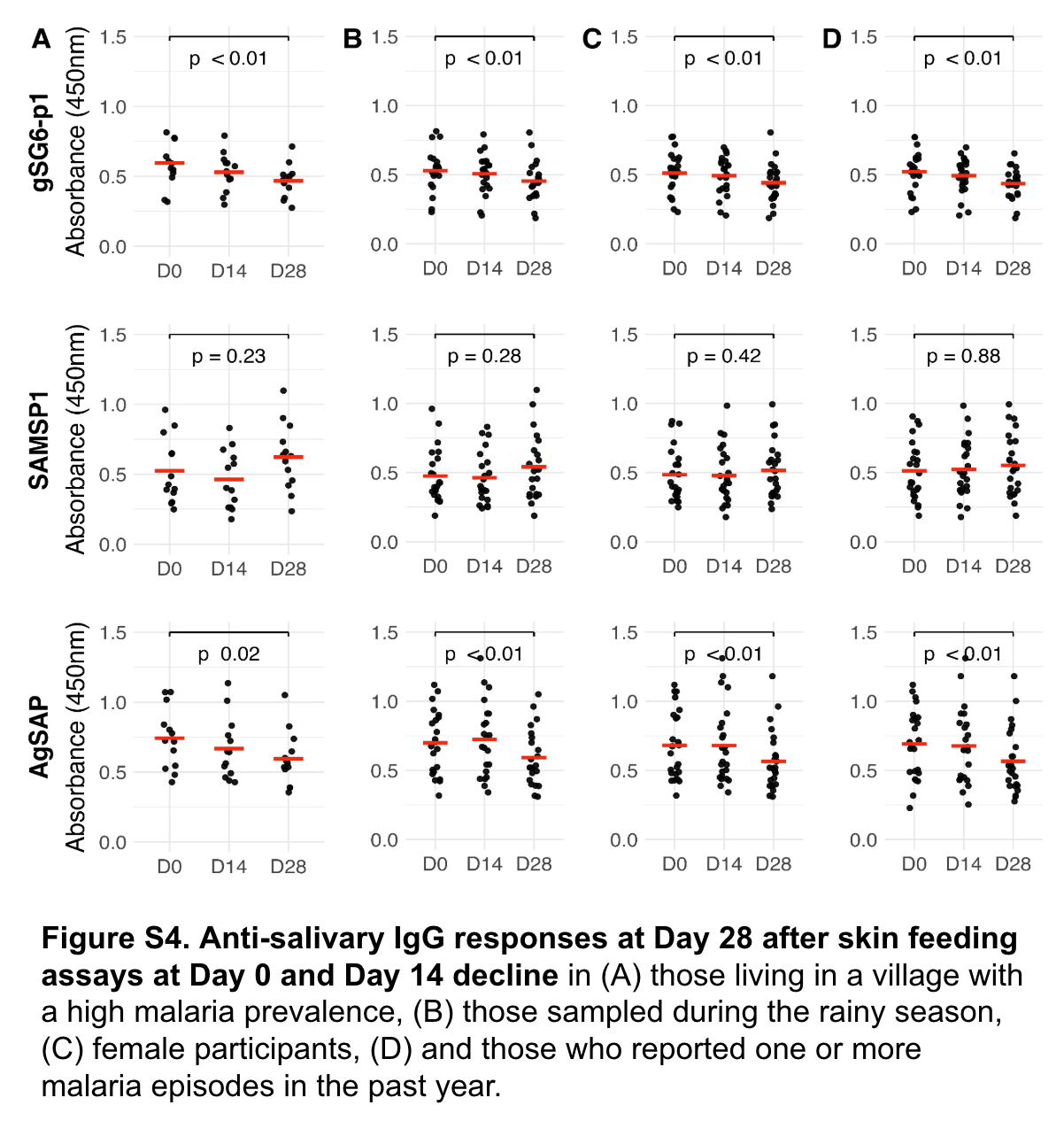
**

**
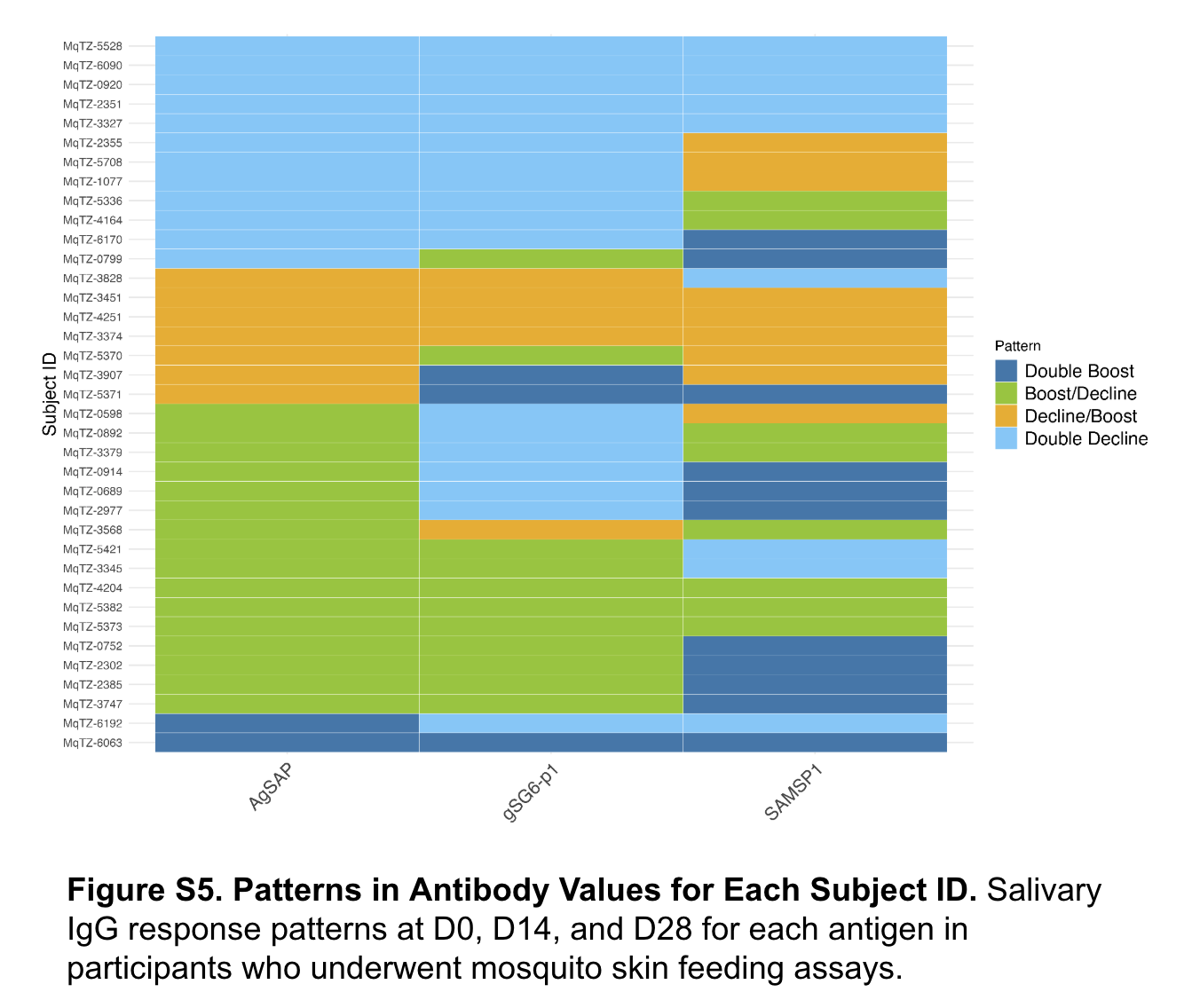
**

Supplement: Supplementary file 1 — Supplementary material 1. [file 13071_2025_7072_MOESM1_ESM.docx]
